# Application of Cerebrospinal Fluid Host Protein Biosignatures in the Diagnosis of Tuberculous Meningitis in Children from a High Burden Setting

**DOI:** 10.1155/2019/7582948

**Published:** 2019-04-16

**Authors:** Charles M. Manyelo, Regan S. Solomons, Candice I. Snyders, Portia M. Manngo, Hygon Mutavhatsindi, Belinda Kriel, Kim Stanley, Gerhard Walzl, Novel N. Chegou

**Affiliations:** ^1^DST-NRF Centre of Excellence for Biomedical Tuberculosis Research, South African Medical Research Council Centre for Tuberculosis Research, Division of Molecular Biology and Human Genetics, Faculty of Medicine and Health Sciences, Stellenbosch University, Po Box 241, Cape Town 8000, South Africa; ^2^Department of Paediatrics and Child Health, Faculty of Medicine and Health Sciences, Stellenbosch University, Po Box 241, Cape Town 8000, South Africa

## Abstract

**Background:**

The diagnosis of tuberculous meningitis (TBM) especially in children is challenging. New tests are urgently needed for the diagnosis of the disease, especially in resource-limited settings.

**Methods:**

We collected cerebrospinal fluid (CSF) samples from children presenting with symptoms requiring investigation for meningitis at a tertiary hospital in Cape Town, South Africa. Children were later classified as TBM or no TBM using published case definitions. Using a multiplex platform, we investigated the concentrations of biomarkers comprising a previously established 3-marker biosignature (VEGF, IL-13, and LL-37) and other potentially useful host biomarkers as diagnostic candidates for TBM.

**Findings:**

Out of 47 children, age, 3 months to 13 years, 23 were diagnosed with TBM and six (16%) were HIV-infected. We validated the previously identified CSF biosignature (sensitivity of 95.7% (95% CI, 79.0-99.2%) and specificity of 37.5% (95% CI, 21.2-57.3%)). However, substitution of IL-13 and LL-37 with IFN-*γ* and MPO, respectively, resulted in improved accuracy (area under the ROC curve (AUC) = 0.97, 95% CI, 0.92-1.00, up to 91.3% (21/23) sensitivity and up to 100% (24/24) specificity). An alternative four-marker biosignature (sICAM-1, MPO, CXCL8, and IFN-*γ*) also showed potential, with an AUC of 0.97.

**Conclusion:**

We validated a previously identified CSF biosignature and showed that refinement of this biosignature by incorporation of other biomarkers diagnosed TBM with high accuracy. Incorporation of these biomarkers into a point-of-care or bedside diagnostic test platform may result in the improved management of TBM in children.

## 1. Introduction

Tuberculosis (TB) remains a global health problem and was responsible for the deaths of estimated 1.6 million people in 2017 [1]. Although it is difficult to accurately estimate the burden of childhood TB, one million children were estimated to have become ill with the disease in 2017 [1].

Tuberculous meningitis (TBM) is the most severe form of extra pulmonary TB as it affects the central nervous system (CNS). It mostly occurs during early childhood and has high morbidity and mortality, mainly due to delayed diagnosis [2]. It is well established that diagnosing pulmonary TB disease in children is challenging, especially in resource-constrained settings [3]. It is even more challenging to diagnose extrapulmonary TB in this patient group, including TBM. As a consequence, TBM frequently results in a poor outcome due to nonspecific symptoms and signs [4]. The limitations of both the most widely used diagnostic test for TB (smear microscopy) [5] and the culture gold standard test are well publicised [3, 5]. These tests have been shown to be suboptimal in the diagnosis of TBM [6, 7]. The GeneXpert MTB/RIF test®, the most important recent advancement in the field of TB diagnostics, is limited by the large CSF volumes required and low negative predictive value [8]. However, the use of the GeneXpert Ultra resulted in an improved negative predictive value in a more recent study on HIV-positive adults [9]. The diagnosis of TBM is mostly based on a combination of clinical findings, multiple laboratory tests on the CSF, imaging findings, and the exclusion of common differential diagnoses in routine clinical practice [10]. Diagnosing the disease in most high-burden, but resource-constrained settings is difficult due to the unavailability of most of these techniques, with children seen at primary and secondary healthcare facilities often having multiple missed opportunities; up to six visits before the eventual diagnosis of TBM are made [11]. New tests are urgently needed for the diagnosis of TBM.

Host biomarker-based tests may be valuable in the diagnosis of TBM as they have previously been shown to be potentially useful in other extrapulmonary forms of TB [12] and may be easily converted into point-of-care or bedside tests [13, 14]. In a previous study, we identified a 3-marker CSF host protein biosignature comprising VEGF, IL-13, and cathelicidin LL-37, which showed potential in the diagnosis of TBM in children [15]. As there have been multiple recent studies in which new, potentially useful TB diagnostic biomarkers were identified [16, 17], we hypothesized that at least some of these biomarkers, which were identified in mostly adult pulmonary TB studies, will be useful in the diagnosis of TBM in children. We therefore aimed to evaluate the usefulness of our previously established 3-marker CSF biosignature in a new cohort of children with suspected meningitis and to also evaluate the potential of other host biomarkers that have shown potential in adult pulmonary TB studies, as candidate markers for the diagnosis of TBM in children. We further hypothesized that the accuracy of the previously identified 3-marker CSF signature [15] may be improved if refined through the incorporation of some of these new biomarkers.

## 2. Materials and Methods

Children suspected of having meningitis and requiring CSF examination for routine diagnostic purposes were recruited from the Tygerberg Academic Hospital in Cape Town, South Africa. Study participants were recruited between November 2016 and November 2017. Children were eligible for participation in the study if they were between the ages 3 months and 13 years, provided that written informed consent was obtained from the parents or legal guardians. Assent was obtained from children older than 7 years if they had a normal level of consciousness, i.e., a Glasgow Coma Score (GCS) of 15/15. The study was approved by the Health Research Ethics Committee of the University of Stellenbosch, Tygerberg Academic Hospital, and the Western Cape Provincial Government.

After the collection of CSF and blood samples for routine diagnostic purposes, an additional 1 ml of CSF was collected into a sterile tube (BD Biosciences). Samples were then taken to the immunology research laboratory for further processing for research purposes, within an average of 2 hours from collection. CSF samples were centrifuged in a biosafety level 3 laboratory at 4000 xg for 15 minutes, followed by aliquoting and storage at -80°C until analysed.

### 2.1. Diagnostic Work-Up of Study Participants

All patients underwent a comprehensive clinical evaluation by a specialist paediatric neurologist. After routine clinical investigations, computed tomography (CT) of the brain, air encephalography, and magnetic resonance (MR) imaging were performed as clinically indicated. Following lumbar puncture, investigations including appearance and colour determination, differential cell counts, protein, glucose, and other routinely investigated markers (Supplementary Table 1) were assessed, followed by centrifugation of the CSF, Gram staining, India ink examination, culture of the centrifuged sediment on blood agar plates (for pyogenic bacteria), auramine “O” staining and fluorescence microscopy, culture using the mycobacterium growth indicator tubes (MGIT)™ method (Becton and Dickinson), and examination for *M.tb* DNA using the HAIN Genotype MTBDRplus kit (Hain Lifescience GmbH, Germany). All data generated from the study were recorded in a study-specific RedCap web-based database.

Patients were classified as possible, probable, or definite TBM according to a uniform research case definition based on a scoring system consisting of clinical, CSF, and neuroimaging findings [18]. TBM was classified as “probable” when patients scored ≥12 when neuroimaging was available and ≥10 when neuroimaging was unavailable. A diagnosis of definite TBM was made if acid-fast bacilli were present in the CSF, *M.tb* was isolated from the CSF by cultured, a nucleic acid amplification test of CSF yielded positive results, or there was histopathological evidence of *M.tb* from another CNS sites.

### 2.2. Immunoassays

In addition to the three biomarkers that comprised our previous 3-marker model [15] (IL-13, VEGF, and cathelicidin LL-37), we evaluated the concentrations of 66 other candidate biomarkers including markers that were previously investigated in adult TB studies [16, 17, 19, 20] and other proteins which have not been previously investigated in the TB field, including complement factors and other proteins, as possible diagnostic biomarkers for TBM by ELISA (cathelicidin LL-37) or the Luminex platform (all other host biomarkers).

Cathelicidin LL-37 levels in CSF samples were evaluated using an ELISA kit purchased from Elabscience Biotechnology Inc. (catalog #E-EL-H2438). Experiments were done according to the procedure recommended by the manufacturer after which optical densities (OD) were read at 450 nm by an automated microplate reader (iMark™ Microplate Reader, Bio-Rad Laboratories). The mean OD of the blank wells was subtracted from the OD of the sample wells and the background-corrected ODs used for statistical analysis.

The concentrations of CCL1 (I-309), CCL2 (MCP-1), CCL3 (MIP-1*α*), CCL4 (MIP-1*β*), CD40 ligand (CD40L), CXCL8 (IL-8), CXCL9 (MIG), CXCL10 (IP-10), granulocyte colony-stimulating factor (G-CSF), granulocyte-macrophage colony-stimulating factor (GM-CSF), interferon gamma (IFN-*γ*), interleukin- (IL-) 1*β*, IL-10, IL-12/23 (p40), IL-17A, IL-21, IL-4, IL-6, IL-7, matrix metalloproteinase- (MMP-) 1, MMP-8, transforming growth factor- (TGF-) *α*, tumour necrosis factor- (TNF-) *α*, soluble neural cell adhesion molecule (sNCAM-1/CD56), MMP-7, ferritin, and MMP-9 were evaluated in patient samples using Luminex kits purchased from R&D Systems Inc. (Bio-Techne), Minneapolis, USA. The levels of apolipoprotein- (Apo-) AI, Apo-CIII, complement C3, complement factor H, BDNF, cathepsin D, soluble intracellular adhesion molecule- (sICAM-) 1, myeloperoxidase (MPO), platelet-derived growth factor- (PDGF-) AA, CCL5 (RANTES), PDGF-AB/BB, soluble vascular adhesion molecule (sVCAM-1), plasminogen activator inhibitor-1 (PAI-1) (total), S100 calcium-binding protein B (S100B), amyloid beta 1-40 (Ab40), Ab42, soluble receptor for advanced glycation end products (sRAGE), Glial cell-derived neurotrophic factor (GDNF), C-reactive protein (CRP), alpha-2-antitrypsin (A1AT), pigment epithelium-derived factor (PEDF), serum amyloid P (SAP), CCL18 (MIP-4/PARC), complement C4 (CC4), CC2, CC4b, CC5, CC5a, CC9, complement factor D (CFD/adipsin), mannose-binding lectin (MBL), complement factor 1 (CF1), sP-selectin, von Willebrand factor-cleaving protease (ADAMTS13), D-DIMER, growth differentiation factor- (GDF-) 15, myoglobin, lipocalin 2 (NGAL), and serum amyloid A (SAA) were evaluated using kits purchased from Merck Millipore, Billerica, MA, USA. All biomarkers were evaluated in CSF samples collected from all study participants following the instructions of the respective kit manufacturers (R&D Systems and Merck Millipore), respectively. All experiments were performed on the Bio Plex platform (Bio Rad Laboratories, Hercules, USA) in an ISO 15189 accredited laboratory. Data acquisition and analysis of median fluorescent intensity were done using the Bio Plex Manager version 6.1 software (Bio Rad Laboratories). The laboratory staff performing the Luminex experiments were blinded to the clinical classification of the study participants. The values of analytes in the quality control reagents evaluated with the samples were within their expected ranges.

### 2.3. Statistical Analysis

Differences in the concentrations of host biomarkers between the TBM and the no TBM groups were assessed using the Mann–Whitney *U* test. The receiver operator characteristic (ROC) curve analysis procedure was used to assess the diagnostic accuracy of individual host biomarkers for TBM. Optimal cut-off values and associated sensitivities and specificities were selected based on Youden's index [21]. The utility of combinations of biomarkers in the diagnosis of TBM was ascertained by general discriminant analysis (GDA), followed by leave-one-out cross-validation. The data was analysed using Statistica (TIBCO Software Inc., CA, USA), and GraphPad Prism version 6 (GraphPad software, CA, USA).

## 3. Results

**Figure 1:**
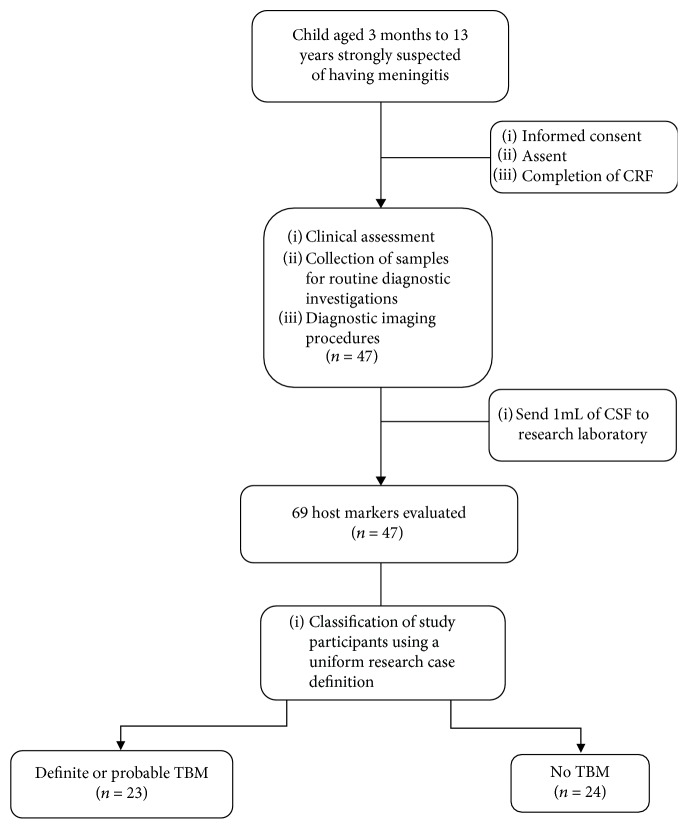
STARD diagram showing the study design and classification of study participants. CRF: case report form; TBM: tuberculous meningitis; no TBM: individuals presenting with symptoms and investigated for TB but TBM ruled out. The no TBM group included bacterial meningitis (*n* = 2), viral meningitis (*n* = 2), and children with other diagnoses (Table 1).

A total of 47 children in whom meningitis was strongly suspected, 30 (63.8%) of whom were males, were included in the study (Figure 1). The median age of all the children was 22 months (interquartile range: 10.5 to 57 months), and six out of 37 with known HIV status (16.2%) were HIV-infected. Using a composite reference standard based on a uniform research case definition of TBM [18], 23 of the children were diagnosed with definite or probable TBM (definite = 2; probable = 21). The 24 children without TBM included children with bacterial meningitis (*n* = 2) and viral meningitis (*n* = 2) and children with other diagnoses (Table 1).

### 3.1. Usefulness of the Previously Identified 3-Marker CSF Biosignature in the Diagnosis of TBM

As we were interested in validating the diagnostic accuracy of the previously established 3-marker CSF biosignature (VEGF, IL-13, and cathelicidin LL-37), we first looked at the utility of individual analytes comprising this signature, followed by the evaluation of combinations between different biomarkers comprising the signature.

As observed in our previous study [15], VEGF was the most useful individual biomarker in this signature as none of the other two markers showed significant differences between groups with the Mann–Whitney *U* test. The median levels of all the three analytes were higher in children with TBM (Table 2). As reagent kits from different manufacturers were used in this study, in comparison with what was employed in the previous study, we performed receiver operator characteristic (ROC) curve analysis to ascertain the optimal threshold values for the analytes using these new reagent kits. Using these new cut-off values, we observed that combining all three biomarkers (that is, a patient yielding positive results with all three), or positivity with any two out of the three analytes, was inferior to the accuracy obtained with VEGF A alone. However, when considering values above the threshold for any one of the three markers taken as a positive result, the accuracy of the 3-biomarker signature improved, with positive and negative predictive values of 59.5% (95% CI, 51.5-66.9%) and 90.0% (95% CI, 55.3-98.5%), respectively (Table 2).

### 3.2. Utility of Alternative Host Biomarkers in the Diagnosis of TBM

**Figure 2:**
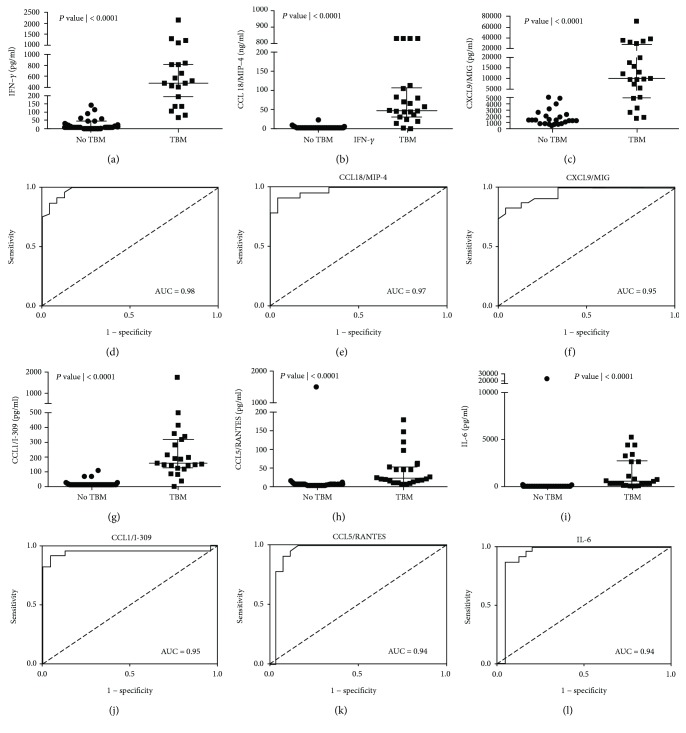
Representative plots showing the concentrations of biomarkers detected in CSF samples from children with and without TBM and ROC curves showing the accuracies of these biomarkers in the diagnosis of TBM. Error bars in the scatter-dot plots indicate the median and interquartile ranges. Representative plots for six analytes with AUC ≥ 0.80 are shown. The accuracies of all host biomarkers evaluated in the study are shown in Supplementary Table 2.

When the concentrations of the other 66 host markers investigated in our study were compared between children with and those without TBM using the Mann–Whitney *U* test, the levels of multiple host biomarkers were significantly different (*p* ≤ 0.05) between the two groups (Supplementary Table 2). When the data for individual host markers were assessed by ROC curve analysis, the area under the ROC (AUC) was above 0.70 for 45 of the 66 proteins. Of note, the AUCs for 28 of these proteins including IFN-*γ*, MIP-4, CXCL9, CCL1, RANTES, IL-6, TNF-*α*, MPO, MMP-9, MMP-8, CC2, IL-10, PAI-1, CXCL8, IL-1b, A1AT, CXCL10, G-CSF, CC4, CC4b, GM-CSF, PDGF AB/BB, Apo-AI, MBL, ferritin, CC5a, SAP, and CC5 were ≥0.80 (Figure 2, Supplementary Table 2).

As all the six known HIV-infected children were in the no TBM group, we excluded these children and reanalysed the data for individual host biomarkers, to assess the possible influence of HIV infection on the accuracy of the biomarkers. After excluding the HIV-infected children, the median levels of PEDF, IL-12/23p40, MMP-1, CD40L, and GDF-15 were no longer significantly different between the children with TBM vs. no TBM. CD40L and GDF-15 showed trends for significant differences (0.05 < *p*-value ≤ 0.09), whereas the observations for all other host markers were unchanged (data not shown).

### 3.3. Utility of Combinations between Other Host Biomarkers in the Diagnosis of TBM

**Figure 3:**
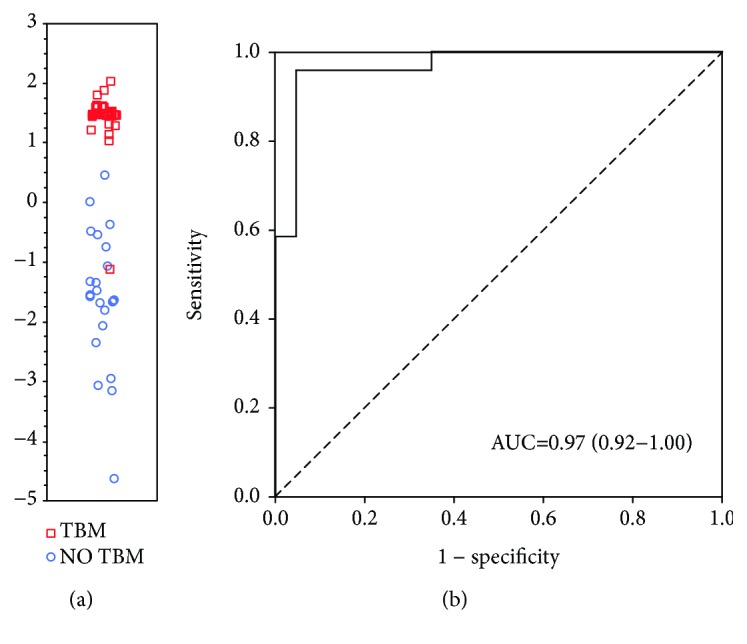
Accuracy of the 4-marker CSF biosignature (sICAM-1, MPO, CXCL8, and IFN-*γ*) in the diagnosis of TBM. Scatter plot showing the ability of the 4-marker signature to classify children as TBM or no TBM (a). ROC curve showing the accuracy of the 4-marker biosignature (b). Red squares: children with TBM. Blue circles: children with no TBM.

When the data obtained for all host markers (including VEGF A, IL-13, and LL-37) were fitted into general discriminant analysis (GDA) models irrespective of HIV status, optimal prediction of TBM was shown to be achieved with a combination of four markers. The most accurate four-marker biosignature is comprised of sICAM-1, MPO, CXCL8, and IFN-*γ* diagnosed TBM with an AUC of 0.97 (95% CI, 0.92-1.00), corresponding to a sensitivity of 87.0% (20/23) (95% CI, 66.4-97.2%) and specificity of 95.8% (23/24) (95% CI, 78.9-99.9%). After leave-one-out cross validation, there was no change in the sensitivity (87.0%) and specificity (95.8%) of the four-marker biosignature. The positive and negative predictive values of the biosignature were 95.2% (95% CI, 74.5-99.3%) and 88.5% (95% CI, 72.7-95.7%), respectively. Further optimization of the four-marker biosignature resulted in both sensitivity and specificity (96%) (Figure 3).

**Figure 4:**
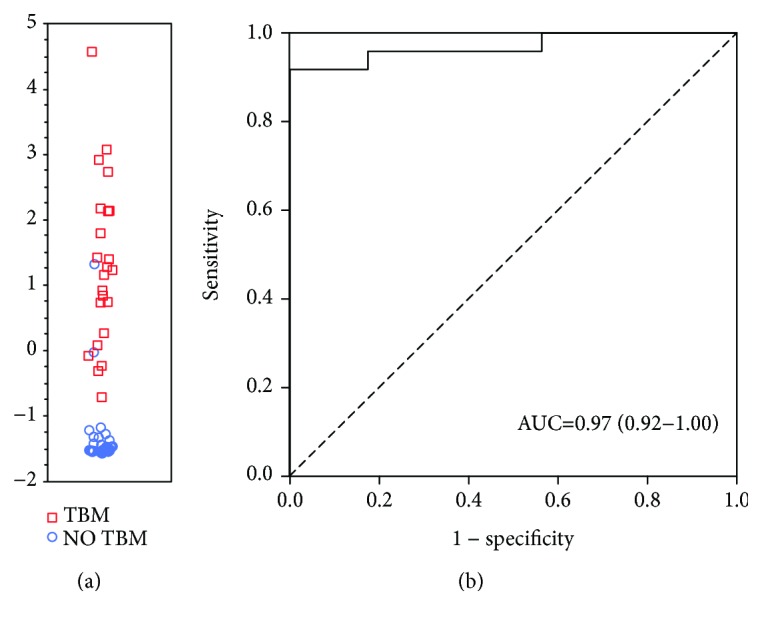
Accuracy of a new VEGF-based 3-marker CSF biosignature in the diagnosis of TBM. Scatter plot showing the ability of the 3-marker signature to classify children as TBM or no TBM (a). ROC curve showing the accuracy of the 3-marker biosignature (VEGF, IFN-*γ*, and MPO) (b). Red squares: children with TBM. Blue circles: children with no TBM.

Given that VEGF has consistently shown promise as a biomarker for TBM [15, 22–24] and that we identified other candidate biomarkers with strong potential in the present study, we wondered whether the previous 3-marker VEGF-based biosignature could be further optimized using other analytes. A GDA model in which IL-13 and cathelicidin LL-37 were replaced with IFN-*γ* and MPO, respectively, resulted in an improved AUC of 0.97 (95% CI, 0.92-1.00), corresponding to a sensitivity of 82.6% (19/23) (95% CI, 61.2-95.1%) and specificity of 95.8% (23/24) (95% CI, 78.9-99.9%). After leave-one-out cross validation, the sensitivity and specificity of the biosignature were 78.3% (18/23) (95% CI, 56.3-92.5%) and 91.7% (22/24) (95% CI, 73.0-99.0%), respectively. The positive and negative predictive values of the refined VEGF-based biosignature after leave-one-out cross validation were 90.0% (95% CI, 70.1-97.2%) and 81.5% (95% CI, 66.8-90.6%), respectively. Further optimization of the biosignature through the selection of better cut-off values resulted in improved sensitivity and specificity of 92% and 100%, respectively (Figure 4).

## 4. Discussion

We assessed the utility of a previously identified 3-marker CSF biosignature (IL-13, VEGF, and cathelicidin LL-37) [15] as well as host biomarkers that have shown potential as pulmonary TB diagnostic candidates in recent adult studies, as tools for the diagnosis of TBM in children with suspected meningitis. Although we validated the diagnostic accuracy of the previously identified 3-marker biosignature, other major findings from our study included the identification of a novel four-marker CSF biosignature comprising sICAM-1, MPO, CXCL8, and IFN-*γ* and a modified 3-marker signature (VEGF, IFN-*γ*, and MPO) which diagnosed TBM with promising accuracy. We also identified multiple host biomarkers that are detectable in CSF and which showed strong potential as diagnostic candidates for TBM in children.

It is well known that the diagnosis of TB disease in children remains a major challenge worldwide. This is mainly due to several well-publicised limitations in the currently available diagnostic tools [3, 5]. It is even more challenging to diagnose extrapulmonary TB including TBM in this patient group, with unstandardized and cumbersome approaches without reliable diagnostic criteria currently being used in routine clinical practice [25, 26]. In order to improve standardization of clinical diagnosis of TBM for research purposes, a uniform research case definition for both adults and children was proposed, categorizing patients as definite, probable, or possible TBM according to a composite score based on clinical, CSF, and neuroimaging findings [18]. None of the tests that are used in the diagnosis of TBM in children performs with high accuracy individually [6, 7, 27, 28]. The disease consequently results in high morbidity and mortality, due mainly to diagnostic delay [2, 29].

Host inflammatory biomarker-based biosignatures have been shown to have potential in the diagnosis of TB disease in both adults and children in previous studies [16, 20, 30]. Furthermore, these immunological biomarker-based tests have the potential to be readily converted into user-friendly, point-of-care diagnostic tests [13, 14] with the development of such tools for the management of TBM especially in children promising to be a major breakthrough in the future. In the present study, we validated the diagnostic accuracy of a previously identified CSF inflammatory biomarker-based biosignature [15]. Although a diagnostic test with positive and negative predictive values of 59.5% and 90.0%, respectively, will be imperfect, such a test may indeed contribute significantly to the management of children with suspected TBM if it is a rapid, point-of-care or bedside test, considering that it currently takes up to six hospital visits before TBM is diagnosed in children with the current diagnostic approaches in a country such as South Africa, which is relatively well resourced, compared to other lower- and middle-income countries [11]. Our data indicates that replacement of two of the proteins in this previously identified signature (IL-13 and cathelicidin LL-37) with new biomarkers (MPO and IFN-*γ*, respectively) has the potential to yield a test with both sensitivity and specificity (>95%). Furthermore, host markers comprising the alternative four-marker biosignature (sICAM-1, MPO, CXCL8, and IFN-*γ*) and other analytes that showed potential individually may be backup host markers that might be employed during the development of such a test. Our findings may therefore pave the way for the development of a prototype CSF biomarker-based test for the diagnosis of TBM in children.

During the development of such a test, the biosignature could be optimized further for use as a rule-in or rule-out test and the newly developed tests used as a screening test for TBM. If the test is based on a point-of-care diagnostic platform, such as the lateral flow technology, successful implementation of the test at the point-of-care or bedside would lead to a significant reduction in the costs and delays that are currently incurred in the diagnosis of TBM in children [11], with a consequent reduction in morbidity and mortality. Although a CSF-based point-of-care or bedside test will be useful in the management of TBM in children, the expertise required for lumbar punctures, opposed to that needed for the collection of other samples such as blood, saliva, or urine, may be a challenge in resource-limited settings, making the implementation of such a diagnostic tool difficult. That notwithstanding, such a test will also contribute to the management of the disease in children where sample collection is possible.

The fact that host inflammatory biomarkers detectable in CSF show potential in the diagnosis of TBM is not surprising, given that previous studies identified VEGF and other candidate biomarkers [15, 22–24] as potential tools for the diagnosis of the disease. Such candidate biomarkers that are detectable in biological fluids including blood [16, 17, 31–33], saliva [19, 20, 34], urine [35], and other specimens [12, 14, 36, 37] have been identified as TB diagnostic candidates in several previous studies. It is well established that individual host biomarkers might not suffice as diagnostic tools for TB disease [16, 31, 33] owing to the fact that these inflammatory biomarkers will be raised in other diseases, including cancers. However, these specificity concerns may be addressed through the use of a panel of biomarkers as done in the present study.

The main limitation of the current study was the relatively small sample size, especially the few children with confirmed TBM and alternative diagnoses including children with other forms of meningitis. However, as this was a TBM-suspect study, the design of the study was relatively strong and the number of participants enrolled into the study is consistent with the patient numbers described in multiple previous studies. Validation of the previously established 3-marker CSF signature in the current study indicates that the novel biosignatures identified in the study have strong potential. Further studies should include larger numbers of study participants with suspected meningitis, including those who are HIV-infected, and individuals with confirmed alternative meningitides. HIV-infected children included in such studies should be appropriately staged with CD4 counts and viral loads, to assess the possible influence of severe HIV infection on the accuracy of the diagnostic biosignatures. For the biosignatures described in the current study to be useful in the management of children with suspected TBM, the biosignatures would require incorporation into a point-of-care or bedside diagnostic test platform, for example, based on the lateral flow technology. Such prototype blood-based TB tests have been developed and successfully investigated in multiple African countries [13, 14], with multibiomarker finger prick-based formats currently under the development for the diagnosis of adult pulmonary TB disease (http://www.screen-tb.eu). Incorporation of host inflammatory biomarkers into such a platform may be relatively easier and faster as lessons learned during the development of adult pulmonary TB tests will be beneficial.

In conclusion, we validated a previously established 3-marker CSF biosignature as a tool for the adjunctive diagnosis of TBM in children and furthermore showed that modification of this signature through the substitution of two of the proteins with new protein biomarkers results in a strong biosignature for the diagnosis of the disease. These biosignatures will only be beneficial for people who would benefit from the urgently required new tools (children with suspected TBM, parents) if these signatures are further validated in other geographical areas and are developed in point-of-care or bedside diagnostic tests. Our study therefore paves the way for the development of such a prototype test.

## Figures and Tables

**Table 1:** Clinical and demographic characteristics of children included in the study.

	All	TBM	No TBM^#^
Number of participants	47	23	24
Median age, months (IQR)	22.0 (10.5-57.0)	18.0 (11.0-40.0)	30.0 (9.0-96.0)
Males, *n* (%)	30 (63.8)	13 (56.5)	17 (70.8)
HIV-positive, *n*/not tested	6/37	0/22	6/15

^#^The no TBM group included children with bacterial meningitis (*n* = 2), viral meningitis (*n* = 2), asphyxia (*n* = 1), autoimmune encephalitis (*n* = 1), febrile seizure (*n* = 3), Guillain Barre (*n* = 1), HIV encephalopathy (*n* = 1), focal seizures (*n* = 1), leukemia (*n* = 1), miliary TB (with lymyphocytic interstitial pneumonitis) (*n* = 1), developmental delay (*n* = 1), breakthrough seizure (*n* = 1), gastroenteritis (caused by shock) (*n* = 1), idiopathic intracranial hypertension (IIH) (*n* = 1), viral gastroenteritis (adeno and rota) and encephalopathy (*n* = 1), stroke (*n* = 1), mitochondrial diagnosis (*n* = 1), viral pneumonia (this included also SAM and nosocomial sepsis) (*n* = 1), febrile seizure and acute gastroenteritis (*n* = 1), and others (*n* = 1). IQR: interquartile range.

**Table 2:** Utility of the previously established 3-marker CSF biosignature in the diagnosis of TBM in a new cohort of children with suspected meningitis. ^∗^Cut-off values shown for VEGF A and IL-13 are in pg/ml. Values shown for cathelicidin LL-37 are the optical densities.

Biosignature	AUC (95% CI)	Cut-off value	Sensitivity % (95% CI)	Specificity % (95% CI)	PPV	NPV
VEGF A	0.81 (0.67-0.94)	>9.4	82.6 (61.2-95.1)	79.2 (57.9-92.9)	79.2 (63.0-89.5)	82.6 (65.6-92.2)
IL-13	0.58 (0.42-0.75)	>524.9	52.2 (30.6-73.2)	66.7 (44.7-84.4)	60.0 (43.0-74.9)	59.3 (46.6-70.8)
^∗^Cathelicidin LL37	0.55 (0.38-0.71)	>0.045	69.6 (47.1-86.8)	50.0 (29.1-70.9)	57.1 (45.1-68.4)	63.2 (45.1-78.2)
VEGF+IL-13+cathelicidin LL37	0.61 (95% CI, 0.50-0.72)	N/A	30.4 (5.6-50.9)	91.7 (74.2-97.7)	77.8 (44.8-93.8)	57.9 (50.6-64.9)
Any two out of the three biomarkers	0.73 (0.60-0.85)	N/A	78.3 (58.1-90.3)	66.7 (46.7-82.0)	69.2 (55.1-80.5)	76.2 (58.4-84.4)
Any one out of the three biomarkers	0.67 (0.56-0.77)	N/A	95.7 (79.0-99.2)	37.5 (21.2-57.3)	59.5 (51.5-66.9)	90.0 (55.3-98.5)

## Data Availability

The Luminex and ELISA data used to support the findings of this study are available from the corresponding author upon request.

## Abbreviations

CSF: Cerebrospinal fluid
CCL1: Chemokine (C-C motif) ligand 1, also abbreviated as I-309
CCL2: Chemokine (C-C motif) ligand 2, also known as monocyte chemoattractant protein- (MCP-) 1
CCL3: Chemokine (C-C motif) ligand 3, also known as macrophage inflammatory protein- (MIP-) 1*α*
CCL4: Chemokine (C-C motif) ligand 4, also known as macrophage inflammatory protein- (MIP-) 1*β*
CCL5: Chemokine (C-C motif) ligand 5, also known as regulated upon activation, normally T-expressed, and presumably secreted (RANTES)
CCL18: Chemokine (C-C motif) ligand 18, also known as macrophage inflammatory protein- (MIP-) 4
CD40L: Cluster of differentiation 40 ligand
CXCL8: C-X-C motif chemokine ligand 8, also known as interleukin-8
CXCL9: C-X-C motif chemokine ligand 9, also known as monokine induced by interferon gamma (MIG)
CXCL10: C-X-C motif chemokine ligand 10, also known as interferon gamma inducible protein 10 (IP-10)
G-CSF: Granulocyte colony-stimulating factor
GM-CSF: Granulocyte-macrophage colony-stimulating factor
IFN-*γ*: Interferon gamma
IL: Interleukin
MMP: Matrix metalloproteinase
TGF: Transforming growth factor
TNF: Tumour necrosis factor
sNCAM-1: Soluble neural cell adhesion molecule-1, also known as cluster of differentiation 56 (CD56)
Apo: Apolipoprotein, e.g., Apo-CIII (apolipoprotein CIII)
CC3: Complement component C3
CFH: Complement factor H
BNDF: Brain-derived neurotrophic factor
sICAM: Soluble intracellular adhesion molecule
MPO: Myeloperoxidase
PDGF: Platelet-derived growth factor
sVCAM: Soluble vascular adhesion molecule
PAI-1: Plasminogen activator inhibitor-1
S100B: S100 calcium-binding protein B
Ab40: Amyloid beta 1-40
sRAGE: Soluble receptor for advanced glycation end products
GDNF: Glial cell-derived neurotrophic factor
CRP: C-reactive protein
A1AT: Alpha-2-antitrypsin
PEDF: Pigment epithelium-derived factor
SAP: Serum amyloid P
CFD: Complement factor D, also known as adipsin
MBL: Mannose-binding lectin
CF1: Complement factor 1
ADAMTS13: von Willebrand factor-cleaving protease
GDF: Growth differentiation factor
NGAL: Neutrophil gelatinase-associated lipocalin, also known as lipocalin 2
SAA: Serum amyloid A
GDA: General discriminant analysis
AUC: Area under the curve.

## Supplementary Materials

Supplementary Materials Supplementary Table 1: detailed clinical and demographic characteristics, CSF laboratory measurements, and neurological findings in children with and without TBM. TBM = tuberculous meningitis, CSF = cerebrospinal fluid, TB = tuberculosis, BCG = Bacille Calmette-Guerin, PCR = polymerase chain reaction, IQR = interquartile range. ^∗^Three of the children in the no TBM group yielded TB-positive culture results from gastric washings. This is a common finding in children with pulmonary TB but without TB meningitis in our high burden setting. Supplementary Table 2: utility of host biomarkers detectable in CSF samples from children with suspected meningitis in the diagnosis of TB meningitis. Median levels (interquartile ranges in parentheses) of all host markers and accuracies in the diagnosis of TBM as determined by ROC curve analysis are shown. Cut-off values and associated sensitivities and specificities were selected based on Youden's index. ^#^Values shown are in ng/ml, and all other analytes are in pg/ml. ^∗^Values shown are absorbance and not concentration values.
