# Supplementary material for: Application of Cerebrospinal Fluid Host Protein Biosignatures in the Diagnosis of Tuberculous Meningitis in Children from a High Burden Setting
Source: Mediators Inflamm. 2019 Apr 16;2019:7582948. doi: 10.1155/2019/7582948 (PMC6501148; doi:10.1155/2019/7582948)
Supplement: Supplementary Materials — Supplementary Table 1: detailed clinical and demographic characteristics, CSF laboratory measurements, and neurological findings in children with and without TBM. TBM = tuberculous meningitis, CSF = cerebrospinal fluid, TB = tuberculosis, BCG = Bacille Calmette-Guerin, PCR = polymerase chain reaction, IQR = interquartile range. ∗Three of the children in the no TBM group yielded TB-positive culture results from gastric washings. This is a common finding in children with pulmonary TB but without TB meningitis in our high burden setting. Supplementary Table 2: utility of host biomarkers detectable in CSF samples from children with suspected meningitis in the diagnosis of TB meningitis. Median levels (interquartile ranges in parentheses) of all host markers and accuracies in the diagnosis of TBM as determined by ROC curve analysis are shown. Cut-off values and associated sensitivities and specificities were selected based on Youden's index. #Values shown are in ng/ml, and all other analytes are in pg/ml. ∗Values shown are absorbance and not concentration values. [file 7582948.f1.pdf]

Supplementary files:

Supplementary Table 1: Detailed clinical and demographic characteristics of children with and without TBM

**Parameter**	**Non TBM (n=24)**	**TBM (n =23)**
Median age, months (IQR)	30 (9-96)	18 (11-40)
Male Gender	17	13
CSF lymphocytes, cells/µL	31.82	66.4
CSF neutrophils, cells/µL	5.87	24.45
CSF protein, g/L	1.14	1.51
CSF glucose, mmol/L	3.01	2.36
Serum glucose, mmol/L	7.63	3.71
Gastric washing TB positive*	3	6
Decreased level of consciousness	7	6
Fever	7	10
Vomiting	5	7
Weight loss	3	7
Seizures	9	9
Cough	7	9
Symptom duration, days	5.6	9.4
TB contact	7	7
BCG done	16	17
Raised intracranial pressure	5	5
Hemiplegia	6	7
HIV positive	6	0
Bateriologically-confirmed TBM	0	2
CSF bacterial culture positive	1	0
Viral PCR positive	2	1
Clear CSF	21	19

TBM= tuberculous meningitis; CSF= cerebrospinal fluid, TB= tuberculosis, BCG= *Bacille Calmette-Guerin;* PCR= polymerase chain reaction; IQR = Inter-quartile range; *three of the children in the Non TBM group yielded TB positive culture results from gastric washings. This is a common finding in children with pulmonary TB but without TB meningitis in our high burden setting.

Supplementary Table 2: Utility of host biomarkers detectable in CSF samples from children with suspected meningitis in the diagnosis of TB meningitis. Median levels (inter-quartile ranges in parenthesis) of all host markers and accuracies in the diagnosis of TBM as determined by ROC curve analysis are shown. Cut-off values and associated sensitivities and specificities were selected based on the Youden's index. #Values shown are in ng/ml, all other analytes are in pg/ml.*Values shown are absorbance and not concentration values.

**Host marker**	**Median in TBM (IQR)**	**Median in No-TBM (IQR)**	**p-value**	**AUC (95% CI)**	**Cut-off Value**	**Sensitivity % (95% CI)**	**Specificity % (95% CI)**
VEGF	45.4 (15.1-150.2)	3.1 (2.5-8.1)	0.000300	0.81 (0.67-0.94)	>9.4	82.6 (61.2-95.1)	79.2 (57.9-92.9)
IL-13	671.68 (246.08-1409.68)	378.2 (89.0-870.4)	0.322220	0.58 (0.42-0.75)	>524.9	52.2 (30.6-73.2)	66.7 (44.7-84.4)
*Cathelicidin-LL37	0.1 (0.1-0.1)	0.0 (0.1-0.1)	0.619521	0.55 (0.38-0.71)	>0.045	69.6 (47.1-86.8)	50.0 (29.1-70.9)
IFN-γ	469.9 (194.0-818.1)	10.3 (3.9-45.7)	<0.0001	0.98 (0.95-1.00)	>99.5	91.3 (72.0-98.9)	91.7 (73.0-99.0)
#MIP-4	47.5 (31.0-105.4)	0.3 (0.2-0.8)	0<0.0001	0.97 (0.94-1.00)	>11.4	91.3 (72.0-98.9)	95.8 (78.9-99.9)
MIG/CXCL9	9846.2 (4983.6-29684.1)	1349.7 (929.7-2205.9)	<0.0001	0.95 (0.90-1.00)	>4855.0	82.6 (61.2-95.0)	95.8 (78.9-99.9)
I-309/CCL1	156.6 (127.2-318.9)	5.4 (3.8-11.4)	<0.0001	0.95 (0.87-1.00)	>74.6	91.3 (72.0-98.9)	95.8 (78.9-99.9)
RANTES	22.3 (14.6-52.0)	3.8 (0.1-5.7)	<0.0001	0.95 (0.87-1.00)	>9.9	91.3 (72.0-98.9)	91.7 (73.0-99.0)
IL-6	524.8 (196.3-2659.9)	2.8 (1.1-12.3)	<0.0001	0.95 (0.88-1.00)	>100.7	87.0 (66.4-97.2)	95.8 (78.9-99.9)
TNF-α	69.2 (50.9-137.6)	1.2 (0.0-8.5)	<0.0001	0.93 (0.85-1.00)	>19.8	95.7 (78.1-99.9)	87.5 (67.6-97.3)

MPO	62078.8 (49640.6-73505.9)	1430.5 (495.4-5436.1)	<0.0001	0.93 (0.83-1.02)	>25823.0	95.7 (78.1-99.9)	91.7 (73.0-99.0)
MMP-9	4074.6 (2081.8-7163.1)	8.6 (0.0-198.8)	<0.0001	0.91 (0.81-1.00)	>963.9	95.7 (78.1-99.9)	91.7 (73.0-99.0)
MMP-8	8640.1 (2811.4-23467.6)	257.3 (0.0-1075.2)	0.000002	0.91 (0.82-1.00)	>1695.0	91.3 (72.0-98.9)	83.3 (62.6-95.3)
#CC2	2188.4 (1229.9-180000.0)	87.5 (41.8-558.7)	0.000006	0.89 (0.78-0.99)	>712.0	87.0 (66.4-97.2)	83.3 (62.6-95.3)
PAI-1	6090.8 (2456.8-12786.0)	401.6 (194.4-1189.3)	0.000008	0.88 (0.77-0.99)	>2163.0	82.6 (61.2-95.1)	87.5 (67.6-97.3)
IL-1β	47.9 (24.2-64.3)	0.0 (0.0-9.7)	0.000009	0.87 (0.76-0.99)	>12.9	82.6 (61.2-95.1)	79.2 (57.9-92.9)
IL-8/CXCL8	970.6 (519.7-1550.8)	110.2 (50.2-331.2)	0.000010	0.88 (0.77-0.99)	>394.8	87.0 (66.4-97.2)	79.2 (57.9-92.9)
IP-10/CXCL10	44900.0 (2102.7-44900.0)	257.6 (85.5-837.4)	0.000016	0.86 (0.75-0.97)	>1200.0	95.7 (78.1-97.2)	79.2 (57.9-92.9)
#A1AT	2209.8 (916.0-6488.9)	338.4 (236.8-866.1)	0.000023	0.87 (0.75-0.98)	>715.3	91.3 (72.0-98.9)	75.0 (53.3-90.2)
IL-10	47.8 (22.2-82.4)	5.3 (0.0-12.0)	0.000037	0.88 (0.76-1.00)	>15.3	91.3 (72.0-98.9)	87.0 (67.6-97.3)
G-CSF	400.2 (178.1-561.0)	0.0 (0.0-152.9)	0.000043	0.85 (0.73-0.97)	>137.5	91.3 (72.0-98.9)	75.0 (53.3-90.2)
#CC4	1201.2 (667.0-2196.0)	336.2 (232.7-593.2)	0.000060	0.84 (0.73-0.96)	>653.3	78.3(56.3-92.5)	79.2 (57.9-92.9)
#CC4b	565.7 (377.5-668.8)	172.7 (94.3-331.7)	0.000103	0.83 (0.71-0.96)	>364.7	78.3 (56.3-92.5)	79.2 (57.9-92.9)

GM-CSF	88.9 (64.7-105.1)	27.9 (13.4-60.5)	0.000112	0.81 (0.71-0.95)	>63.8	78.3 (56.3-92.5)	79.2 (57.9-92.9)
#Apo AI	1708.0 (980.1-7429.6)	150.8 (0.0-980.1)	0.000167	0.82 (0.69-0.95)	>365.4	91.3 (72.0-98.9)	70.8 (48.9-87.4)
CC5a	66.7 (35.1-93.0)	6.4 (4.4-42.8)	0.000173	0.81 (0.68-0.95)	>26.0	82.6 (61.2-95.1)	70.8 (48.9-87.4)
PDGF-AB/BB	12.9 (5.8-24.5)	5.0 (0.9-7.0)	0.000175	0.82 (0.69-0.95)	>7.7	69.6 (47.1-86.8)	87.5 (67.6-97.3)
#MBL	12.3 (3.7-56.2)	1.0 (0.6-6.6)	0.000221	0.81 (0.69-0.94)	>2.9	87.0 (66.4-97.2)	66.7 (44.7-84.4)
Ferritin	4697.7 (3261.4-300000.0)	705.7 (325.5-3376.9)	0.000236	0.81 (0.68-0.94)	>2729.0	91.3 (72.0-98.9)	75.0 (53.3-90.2)
#CC5	344.7 (166.1-724.3)	36.2 (20.8-178.0)	0.000309	0.81 (0.67-0.94)	>155.4	82.6 (61.2-95.1)	75.0 (53.3-90.2)
#SAP	63.4 (34.6-184.6)	9.6 (5.7-33.5)	0.000364	0.81 (0.67-0.95)	>30.8	87.0 (66.4-97.2)	75.0 (53.3-90.2)
#CFH	1242.8 (669.1-5717.9)	238.9 (82.8-795.3)	0.000629	0.79 (0.66-0.93)	>850.9	73.9 (51.6-89.8)	78.3 (56.3-92.5)
ICAM-1	2128.5(1610.6-4313.7)	499.8 (319.5-1190.1)	0.000743	0.79 (0.65-0.93)	>1372.0	82.6 (61.2-95.1)	79.2 (57.9-92.9)
#P-Selectin	1.2 (0.0-1.8)	0.0 (0.0-0.0)	0.001036	0.76 (0.62-0.89)	>0.3	73.9 (51.6-89.8)	83.3 (62.6-95.3)
PDGF-AA	13.6 (7.3-19.9)	5.5 (3.5-7.8)	0.001047	0.78 (0.64-0.92)	>6.6	82.6 (61.2-95.1)	75.0 (53.3-90.2)
TGF-α	10.0 (5.8-25.7)	3.7 (0.0-7.3)	0.001048	0.78 (0.65-0.92)	>8.6	73.9 (51.6-89.8)	83.3 (62.6-95.3)

#NGAL	77.8 (16.8-512.8)	1.7 (0.7-7.3)	0.001401	0.78 (0.61-0.94)	>16.8	78.3 (56.3-92.5)	95.8 (78.9-99.9)
#CC3	886.7 (357.8-1722.5)	192.8 (56.5-749.1)	0.002344	0.76 (0.62-0.91)	>528.6	73.9 (51.6-89.8)	69.6 (47.1-86.8)
MIP-1β/CCL4	356.3 (240.6-624.8)	185.9 (122.5-261.6)	0.002480	0.76 (0.62-0.90)	>261.6	69.6 (47.1-86.8)	75.0 (53.3-90.2)
IL-17A	14.9 (4.9-32.5)	0.0 (0.0-9.2)	0.002642	0.75 (0.60-0.89)	>2.6	82.6 (61.2-95.1)	66.7 (44.7-84.4)
#CRP	230000.0 (522.0-230000.0)	361.6 (64.1-230000.0)	0.003122	0.74 (0.60-0.87)	>116193.43	69.6 (47.1-86.8)	70.8 (48.9-87.4)
NCAM	30138.4 (18759.6-35617.2)	41021.7 (31229.8-52874.4)	0.003673	0.75 (0.61-0.89)	<36722.0	78.3 (56.3-92.5)	66.7 (44.7-84.4)
#CC9	43.1 (35.5-59.0)	27.3 (20.7-35.8)	0.004645	0.74 (0.59-0.90)	>36.6	73.9(51.6-89.8)	83.3 (62.6-95.3)
CD40L	471.7 (350.8-823.8)	263.7 (160.1-426.2)	0.006422	0.73 (0.58-0.88)	>369.6	73.9 (51.6-89.8)	75.0 (53.3-90.2)
#CF1	480.3 (246.4-970.6)	111.3 (83.2-369.5)	0.006448	0.73 (0.58-0.88)	>263.4	73.9 (51.6-89.8)	70.8 (48.9-87.4)
MIP-1α/CCL3	277.3 (208.8-348.8)	179.0 (35.1-262.5)	0.007518	0.73 (0.58-0.87)	>223.9	69.6 (47.1-86.8)	70.8 (48.9-87.4)
#D-dimer	98000.0 (1425.0-98000.0)	95.7 (2.5-1581.2)	0.007729	0.72 (0.56-0.87)	>49857.4	73.9 (51.6-89.8)	79.2 (57.9-92.9)
#Apo CIII	69.8 (22.7-442.0)	14.2 (6.5-48.4)	0.015650	0.71 (0.56-0.87)	>26.3	73.9 (51.6-89.8)	69.6 (47.1-86.8)
VCAM-1	119507.9 (45091.2-149043.0)	41549.9 (17719.0-122798.7)	0.020942	0.70 (0.55-0.85)	>79387.1	69.6 (47.1-86.8)	66.7 (44.7-84.4)

IL-12/23p40	249.0 (0.00-695.8)	0.0 (0.0-181.8)	0.018510	0.69 (0.54-0.84)	>168.7	69.6 (47.1-86.8)	75.0(53.3-90.2)
#Adipsin/Complement factor D	50.1 (37.6-168.9)	26.1 (15.8-64.7)	0.046258	0.67 (0.51-0.83)	>35.5	82.6 (61.2-95.1)	62.5(40.6-81.2)
#GDF-15	0.4 (0.2-0.5)	0.0 (0.0-0.2)	0.049282	0.67 (0.50-0.84)	>0.2	73.9 (51.6-89.8)	79.2 (57.9-92.9)
#PEDF	746.8 (667.8-837.2)	658.0 (575.6-819.8)	0.056793	0.66 (0.50-0.83)	>689.6	73.9 (51.6-89.9)	62.5 (40.6-81.2)
MMP-1	448.8 (328.8-1058.9)	308.9 (243.0-581.1)	0.058111	0.66 (0.50-0.82)	>318.9	78.3 (56.3-92.5)	58.3 (36.6-77.9)
#SAA	450.4 (1.5-230000.0)	6.5 (0.1-254.2)	0.059259	0.66 (0.50-0.82)	>204.9	60.9 (38.5-80.3)	75.0 (53.3-90.2)
Aβ40	580.0 (305.1-918.5)	800.5 (323.6-2195.3)	0.190601	0.61 (0.44-0.78)	<759.5	65.2 (42.7-83.6)	58.3 (36.6-77.9)
#ADMTS13	8.1 (6.3-15.9)	6.1 (0.5-9.7)	0.212312	0.60 (0.44-0.77)	>6.2	78.3 (56.3-92.5)	54.2 (32.8-74.5)
Aβ42	172.8 (54.3-288.2)	219.0 (81.9-645.3)	0.259233	0.60 (0.43-0.76)	<292.1	78.3 (56.3-92.5)	41.7 (22.1-63.4)
#Myoglobin	0.5 (0.1-1.1)	0.1 (0.0-0.9)	0.266718	0.60 (0.43-0.76)	>0.2	73.9 (51.6-89.8)	58.3 (36.6-77.9)
MCP-1/CCL2	812.5 (457.9-1348.7)	1076.2 (513.2-1423.7)	0.292145	0.59 (0.42-0.76)	<881.0	60.9 (38.5-80.3)	66.7 (44.7-84.4)
S100B	41.2 (41.2-2800.0)	41.2 (28.0-64.6)	0.311691	0.59 (0.42-0.77)	>64.6	38.9 (17.3-64.3)	75.0 (50.9-91.3)
MMP-7	101.5 (81.6-181.6)	101.5 (81.6-121.5)	0.329877	0.58 (0.42-0.75)	>111.5	43.5 (23.2-65.5)	70.8 (48.9-87.4)

IL-4	162.6 (107.9-229.2)	191.6 (132.7-248.9)	0.394463	0.57 (0.40-0.74)	<181.1	65.2 (42.7-83.6)	58.3 (36.6-77.9)
sRAGE	14.1 (12.8-15.3)	14.4 (12.8-16.6)	0.473024	0.56 (0.39-0.73)	<14.4	56.5 (34.5-76.8)	50.0 (29.1-70.9)
Cathepsin D	75722.1 (61184.2-91429.2)	66539.1 (50433.7-96857.8)	0.537070	0.55 (0.38-0.72)	>68062.2	69.6 (47.1-86.8)	54.2 (32.8-74.5)
IL-7	4.3 (0.0-7.0)	5.3 (2.4-7.9)	0.645745	0.54 (0.37-0.71)	<4.3	52.2 (30.6-73.2)	58.3 (36.6-77.9)
BDNF	0.6 (0.0-1.1)	0.5 (0.0-1.0)	0.674752	0.54 (0.37-0.70)	>0.5	60.9 (38.5-80.3)	50.0 (29.1-70.9)
IL-21	43.2 (12.7-78.2)	46.8 (30.9-61.1)	0.781809	0.52 (0.35-0.69)	<37.36	47.8 (26.8-69.4)	66.7 (44.7-84.4)
GDNF	2.2 (1.8-2.3)	2.1 (1.9-2.5)	0.828794	0.48 (0.31-0.65)	<2.1	47.8 (26.8-69.4)	41.7 (22.1-63.4)
